# Mucin Gene Expression in Reflux Laryngeal Mucosa: Histological and *In Situ* Hybridization Observations

**DOI:** 10.1155/2014/264075

**Published:** 2014-03-24

**Authors:** Mahmoud El-Sayed Ali, David M. Bulmer, Peter W. Dettmar, Jeffrey P. Pearson

**Affiliations:** ^1^Department of Otolaryngology, Mansoura University Hospital, Mansoura University, Egypt; ^2^Institute for Cell and Molecular Biosciences, Newcastle University, Faculty of Medical Sciences, Newcastle upon Tyne NE2 4HH, UK; ^3^Castle Hill Hospital, Castle Road, Cottingham, East Yorkshire HU16 5JQ, UK

## Abstract

*Objectives/Hypothesis*. To determine if laryngopharyngeal reflux alters mucin gene expression in laryngeal mucosa. *Methods*. *In situ* hybridization was employed to study the expression of the 8 well-characterised mucin genes MUC1-4, 5AC, 5B, 6, and 7 in reflux laryngeal mucosa from laryngeal ventricles, posterior commissures, and vocal folds compared to control/normal laryngeal mucosa. *Results*. MUC1-5 genes are expressed in normal and reflux laryngeal mucosa. MUC1, 3 and 4 are expressed in respiratory and squamous mucosa whereas MUC2 and 5AC are expressed in respiratory mucosa only. MUC3, 4 and 5AC are downregulated in reflux mucosa. MUC5AC expression is significantly reduced in the 3 mucosal sites and when mucosal type was taken into account, this remains significant in combined laryngeal and ventricular mucosa only. *Conclusions*. MUC3, 4 and 5AC expression is downregulated in laryngopharyngeal reflux. This may be due to laryngeal mucosal metaplasia and/or alteration of mucin gene expression in the preexisting mucosa. Altered mucin gene expression might predispose laryngeal mucosa to the damaging effect of reflux.

## 1. Introduction

Mucin gene expression is tissue specific in order to afford protection for the relevant mucosa. In certain conditions such as inflammation, metaplasia, and neoplasia, mucin gene expression patterns can be altered through changes in the nature of the mucosal tissue. An example is Barrett's oesophageal metaplasia when lower oesophageal mucosa changes from squamous to gastrointestinal mucosa containing mucus secreting cells [1]. This appears to result from repetitive insult to oesophageal mucosa by gastric refluxate leading to altered mucin expression from normal membrane-bound mucins and submucosal gland secretory mucins to the secretory gel-forming mucins similar to those found in the gastrointestinal tract. MUC2 expression found in Barrett's metaplasia is lost as the cells become dysplastic [2].

Laryngopharyngeal reflux (LPR) has become more commonly attributed as an etiology of many upper airway disorders for which there has previously been no known aetiology. The anecdotal evidence as to the increased laryngeal mucus noted in reflux patients during laryngoscopy has not been fully elucidated. It is not known whether this increased mucus is laryngeal or tracheobronchial in origin and whether it is due to increased mucus expression or reduced mucus clearance. Although mucin expression in laryngeal cancers has been studied before [3–5], the effect of LPR on laryngeal mucin gene expression has been documented in only a few studies [6, 7]. Furthermore, mucin gene expression in different regions of the laryngeal mucosa has not been studied in detail.

## 2. Aim of Study

This study aims to investigate mucin gene expression in laryngeal mucosa of LPR patients in contrast to control/normal laryngeal mucosa.* In situ* hybridization was employed as it offers not only high specificity but also provides cellular expression details in histological sections. The study includes the first 8 mucin genes, MUC1-4, 5AC, 5B, 6 and 7, the best characterized mucin genes so far.

## 3. Methods

### 3.1. Control Laryngeal Mucosa

Clinically and histo-pathologically normal human laryngeal mucosa was obtained from 3 nonsmoker patients with no documented history of LPR. The first 2 patients had ischemic heart disease. The third patient had localised laryngeal cancer and mucosal samples were taken well away from the cancer. Mucosal samples were obtained from 3 anatomical locations: vocal folds, laryngeal ventricles and posterior commissure as these are the areas often involved in reflux laryngeal disorders.

### 3.2. Reflux Laryngeal Mucosa

Laryngeal mucosal samples from patients with LPR were donated by Professor Koufman, Wake Forest Medical School, NC, USA. All tissues were obtained in accordance with ethical guidelines, with informed consent obtained for each sample, and the study was approved by the institutional review board of Newcastle University. Mucosal samples were taken from the vocal folds, laryngeal ventricles and posterior commissure during micro-laryngoscopic examination. Three samples were taken from a total of 27 LPR patients. Due to the small size of some of the laryngeal mucosal samples, the numbers of samples from the 3 anatomical sites were not equal.

### 3.3. *In Situ* Hybridization

The protocol followed a modified version of that of Aust et al. [8] using 48 bp Oligonucleotide probes with sequences complimentary to the most frequently occurring base-pair sequences within the tandem repeat domain of the mucin mRNA. This was to obtain signal amplification by hybridizing the largest number of probes with the tandem repeat regions in the same mRNA molecule. Positive control tissues were obtained from tissues known to strongly express the investigated mucin gene. Human breast tissue was used as a control for MUC1; human colon for MUC2, 3 and 4; human gastric mucosa for MUC5AC and 6; human bronchial mucosa for MUC5B, and human submandibular salivary gland for MUC7. Negative controls consisted of sections from normal human liver, as it does not express any of these mucin genes. Steps of the experiment were detailed elsewhere [9]. Whenever possible, all mucin genes were tested on the available samples and duplicates were carried out as tissue allowed.

Sections were lightly counterstained in Harris' haematoxylin before immersing in Scott's modified tap water (bluing reagent) and mounted using gelatin and a coverslip. Light microscopy was performed on sections using Nikon Labophot microscope fitted with a trinocular mount and photographed using an Olympus Camedia C-3030 Zoom digital camera at magnification of 100X and 200X. Image clarity was enhanced by adjusting brightness, hue, and contrast with Adobe Photoshop software (Adobe Systems, Mountain view, CA). Histological details were also observed in each slide before looking for the hybridization signals. Positive signal were identified as an intense blue/black darkening in the cytoplasm of cells.* In situ* hybridization data was analysed by a chi square test comparing the two subsets of data and the significance level was considered at *P* < 0.05.

## 4. Results

### 4.1. Histological Observations

In control laryngeal mucosa, posterior commissures were covered by mixed (respiratory and squamous) mucosa in 2/3 of samples and in 1/3 the covering mucosa was squamous epithelium. Vocal cord mucosa was mixed (respiratory and squamous) in the 3 samples whereas ventricular mucosa was covered by respiratory epithelium only. Squamous metaplasia was noted in reflux laryngeal mucosa from the 3 locations. Reflux vocal cord and posterior commissure mucosa was predominantly covered by squamous mucosa in 2/3 of samples and the other 1/3 was covered by mixed epithelium. Mixed mucosa appeared in 23% of ventricular mucosal samples and the remaining 77% was still covered by respiratory epithelium (Table 1).

### 4.2. Mucin Gene Expression in Control Laryngeal Mucosa (Figure 1)

Control laryngeal mucosa expressed MUC1-4 and 5AC depending on the type of mucosa. Thus, the secretory mucin genes MUC2 and 5AC were present only in respiratory mucosa of the ventricles and vocal folds and were absent in squamous mucosa of the posterior commissure and vocal cords. MUC1, 3 and 4 were expressed in both mucosal types. MUC4 was the most prevalent mucin gene expressed in 78% of samples (7/9 of samples) followed by MUC3 and 5AC (67% each). MUC1 and 2 were less prevalent (11% each) (Table 2). MUC6 and 7 were not expressed in any of the control laryngeal samples. MUC5B results were not explored as the positive control for this mucin gene (human bronchial mucosa) consistently failed to show positive expression.

### 4.3. Mucin Gene Expression in Reflux Laryngeal Mucosa (Figure 2)

MUC6 and 7 were not expressed in any of the tested (vocal folds, laryngeal ventricles and posterior commissure) samples. MUC3 and 4 were downregulated (52% in LPR mucosa versus 67% in control mucosa for MUC3 and 69% in LPR mucosa versus 78% in control mucosa for MUC4) whereas MUC1 and 2 were upregulated (21% in LPR mucosa versus 11% in control mucosa for MUC1 and 29% in LPR mucosa versus 11% in control mucosa for MUC2). The only mucin gene which showed significant expression change was MUC5AC which was down regulated in LPR samples from the 3 anatomical sites (16% in LPR mucosa versus 67% in control mucosa) (*P* < 0.001, chi square test) (Table 3).

### 4.4. Topographic Mucin Gene Expression in Reflux Laryngeal Mucosa

Mucin gene expression in individual areas of reflux laryngeal mucosa showed similar patterns as observed in control laryngeal mucosa as a whole.

#### 4.4.1. Posterior Commissure

MUC1 and 2 were expressed in LPR posterior commissure mucosa whereas these 2 mucin genes were not expressed in control mucosa. This however was not statistically significant. MUC4 was slightly up regulated. Although MUC3 and 5AC were down regulated, this was statistically significant only for MUC5AC (*P* < 0.05).

#### 4.4.2. Laryngeal Ventricles (Figure 3)

All the expressed mucin genes were down regulated in the mucosa of laryngeal ventricles to variable extents; however, the only mucin gene which showed significant downregulation was MUC5AC (*P* < 0.001). MUC4 was expressed in only 70% of reflux versus 100% expression in control ventricular mucosa.

#### 4.4.3. Vocal Folds

MUC1 and 2 were expressed in LPR (32% and 14% for MUC1 and 2, resp.) whereas these 2 mucin genes were not expressed in the 3 control vocal cord mucosal samples. However, this was not statistically significant. The other 3 mucin genes MUC3, 4 and 5AC were downregulated although this was not statistically significant.

Secretory mucin expression was analysed taking into account the type of mucosa, that is, the expression of secretory mucin genes by respiratory mucosa only and expression of membrane-bound mucin genes in both mucosal types. This resulted in no difference of MUC2 expression in LPR versus control laryngeal mucosa. MUC5AC down regulation was still significant in respiratory mucosa of the combined laryngeal and ventricular mucosa (*P* < 0.05) while statistical significance was lost in respiratory mucosa of posterior commissure mucosa.

## 5. Discussion

Mucin gene expression in control and reflux laryngeal mucosa is similar to that in other parts of airway mucosa such as tracheal [10], bronchial [11] and nasal mucosa [8, 12]. Although MUC3 is considered as an intestinal mucin gene, it has been found to be expressed in airway mucosa [13, 14] and in nasal polyps [9]. Expression patterns were different among the different laryngeal mucosal sites. This could be accounted for by specific mucin gene expression related to a specific type of mucosa.

There is an overall upregulation of MUC 1 and 2 and down regulation of  MUC3 4 and 5AC mucin gene expression in reflux laryngeal mucosa. Samuels et al. [7] employed RT-PCR to 2 normal and 3 LPR mucosal samples and reported down regulation of MUC2, 3 and 5AC in LPR compared to normal laryngeal mucosa. They found that exposure of normal hypopharyngeal mucosal cell to low pH* in vitro* up regulated these mucins whereas pepsin inhibited this up regulation. They postulated that depletion of mucosal secretions may contribute to the progression of reflux injury. Although the net effect of acid and pepsin was still one of up regulation of MUC2 and 3 and completely abolished up regulation of MUC1 and 5AC in hypopharyngeal cell culture, Samuel et al. postulated that, after repeated exposure to gastric refluxate, pepsin could deregulate the protective stimulation of mucin gene expression and may ultimately lead to the overall down regulation of a subset of mucin genes.

MUC3 and 4, the two main mucin genes controlling the expression of membrane-bound mucins, are down regulated in reflux laryngeal mucosa. The contribution of membrane-bound mucins in laryngeal mucosal protection is unknown. However, the structure of membrane-bound mucins may provide antidesiccating and protective mechanism against passage of air, inhaled particles, vibratory stress (as a result of phonation) and other insults such as contact with noxious refluxed materials. Alteration of this protective mechanism may predispose the laryngeal mucosa to damage and metaplasia.

Secretory mucins MUC2 and 5AC would provide lubricating and protective barrier. MUC2 was expressed only in control ventricular mucosa and was absent in posterior commissure and vocal fold mucosa whereas MUC5AC was moderately expressed in the three locations to various extents. In LPR mucosa, low expression of MUC2 in vocal fold mucosa was associated with moderate expression of MUC5AC and moderate expression of MUC2 in posterior commissure and ventricular mucosa was associated with low expression of MUC5AC. This suggests that respiratory MUC2 and 5AC are not mutually inclusive or exclusive in pathological respiratory mucosa. This is similar to our previous results on sinus mucin expression where an inverse relationship was found between the expression of these two mucins [12]. Airway mucin expression could be contributing to a finite pool, so if for example MUC2 is up regulated, compensatory MUC5AC down regulation results.

MUC5AC was significantly down regulated in LPR mucosa from the ventricular and posterior commissure mucosa not in the vocal fold mucosa. However, when squamous mucosa was excluded from the calculation, the significant reduction of MUC5AC expression in the LPR posterior commissure mucosa was lost. This indicates that MUC5AC down regulation in the posterior commissure mucosa could be, at least in part, due to the replacement of respiratory epithelium by squamous epithelium which is unable to express MUC5AC.

MUC5AC down regulation in laryngeal ventricles was still significant in the respiratory mucosa after exclusion of squamous mucosa. A direct inhibitory effect of reflux on MUC5AC expression independent of squamous metaplasia could be responsible for MUC5AC down regulation in ventricular mucosa. It is to be noted also that respiratory mucosa of laryngeal ventricles demonstrated no total squamous metaplasia in any of the studied samples. The samples which showed squamous metaplasia had areas of respiratory mucosa (mixed mucosa). This was also noted in only 23% of samples in contrast to the posterior commissures and vocal folds where 67% and 64% of samples demonstrated squamous metaplasia and were covered by squamous epithelium only. The low incidence of squamous metaplasia in ventricular mucosa compared to posterior commissures and vocal fold mucosa could be related to the anatomy of the ventricular mucosa being sequestered between ventricular and vocal folds and thus being less likely to be exposed to reflux components. However, this does not agree with the significant down regulation of  MUC5AC expression in reflux ventricular mucosa. An explanation for this could be that the exposure of ventricular mucosa to the reflux insult triggers MUC5AC down regulation as the main insult effect rather than altering the nature of the covering epithelium.

The appearance of squamous metaplasia in an initially respiratory mucosa would decrease the quantity of secretory mucins. It is a possible consequence that any protective effect imparted to the tissue by the expression of these mucins would decrease, so further increasing the susceptibility of the mucosal surface to damage by noxious agents such as reflux components. On the other hand, this squamous metaplasia may indicate another way of tissue defence by creating a multilayer of dead cells which could then incur stronger physical barrier against injurious effect of refluxate. It has been reported that ciliated bronchiolar epithelial cells undergo squamous metaplasia after bronchiolar injury with naphthalene and new squamous cells spread beneath injured epithelial cells maintaining the integrity of the epithelium [15].

Down regulation of membrane-bound mucins MUC3 and 4 would be solely due to direct effect of reflux on laryngeal mucosa as these mucin genes are expressed in respiratory and squamous mucosa. This is in contrast to down regulation of the secretory mucin MUC5AC which could be due to an indirect effect of reflux through squamous metaplasia of laryngeal mucosa in addition to the direct effect on respiratory mucosal mucin gene expression. The possible dual mechanisms of MUC5AC down regulation would explain the significant alteration of this mucin gene in reflux laryngeal mucosa compared to MUC3 and 4. It would also suggest a significant contribution of MUC5AC down regulation in the development of endoscopic laryngeal mucosal changes noted in reflux laryngitis.

Histological sections of laryngeal mucosa did not show submucosal glands. This suggests that MUC5B which is mainly expressed in submucosal gland of airway epithelium [16, 17] might not be a significant member of the laryngeal mucin genes family. This could imply that laryngeal mucosal defence depends mainly on the integrity of the surface mucosa and therefore a change in the nature of laryngeal mucosa could have a significant impact on its protective functions. Therefore, the lack of information about this mucin gene in the current study, due to technical difficulty, does not seem to significantly alter the drawn picture of normal and reflux laryngeal mucin gene expression.

### 5.1. Further Works/Studies Required

Further studies with larger numbers of samples are needed to clarify these observations to create a clearer picture of the impact of reflux on mucin gene expression in laryngeal mucosa. Comparative histological analysis of the spread of respiratory and squamous epithelia in normal versus reflux laryngeal mucosa is important to clarify the possible interplay of laryngeal mucosal changes and mucin gene expression in LPR.

### 5.2. Strength and Weakness of the Study

This study explores mucin gene expression in normal and reflux laryngeal mucosa with some interesting observations. The number of control/normal laryngeal mucosal samples is small. It is a clinical and ethical challenge to get normal laryngeal mucosa from healthy volunteers or patients with nonlaryngeal pathologies. Although clinically normal mucosa can be obtained from laryngectomy samples, subtle changes at the molecular levels may exist and could alter the normal mucin gene expression. Furthermore, laryngeal cancer patients are usually elderly smokers and these, among other factors, could alter mucin gene expression in control laryngeal mucosa.

## 6. Conclusion

LPR tends to downregulate mucin gene expression particularly MUC5AC, a secreted and gel-forming mucin. This change could be due to laryngeal mucosal metaplastic changes and/or alteration of mucin gene expression in the preexisting mucosa. The expression of mucin genes in laryngeal mucosa may offer some protection to laryngeal mucosa and alteration of the mucosal type or the gene expressed may predispose laryngeal mucosa to the damaging effects of reflux. Down regulation of MUC5AC could be involved in the development of reflux-related laryngeal mucosal changes noted in clinical settings.

## Figures and Tables

**Figure 1:**
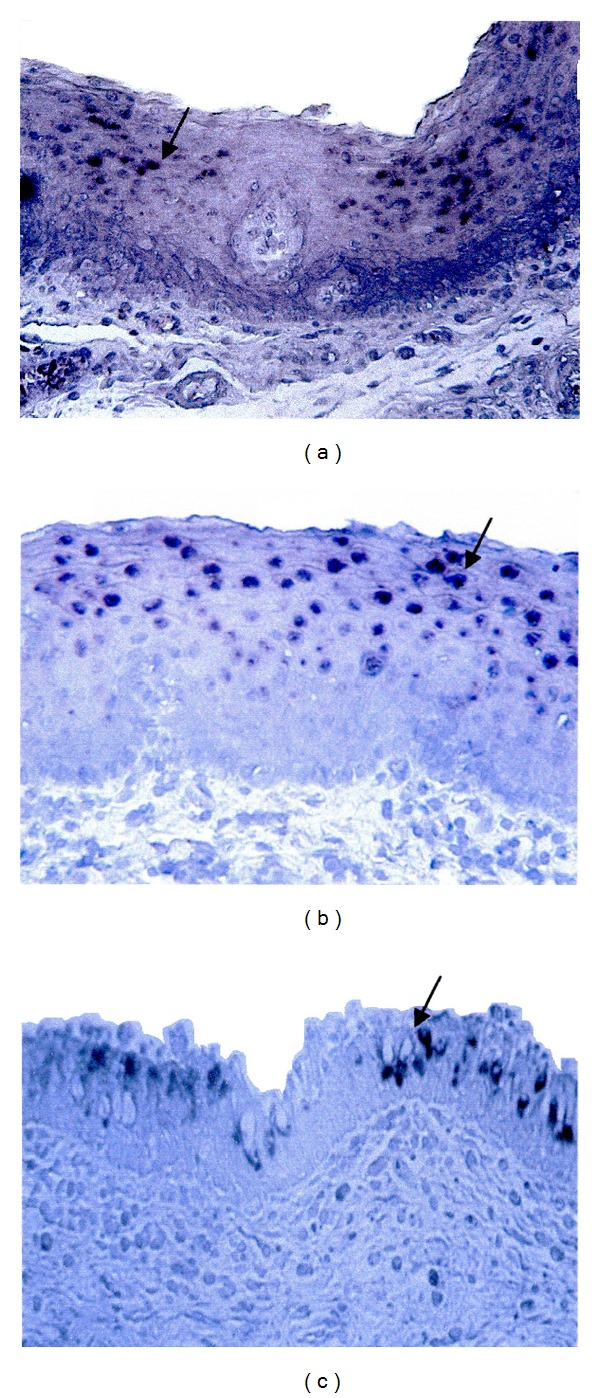
Mucin gene expression in normal laryngeal mucosa.* In situ* hybridization photographs of the expression of MUC3 and 4 in vocal cords mucosa ((a) and (b), resp.) and MUC5AC in laryngeal ventricles mucosa (c). Sections were lightly counterstained in Harris' hematoxylin and then immersed in Scott's modified tap water (bluing reagent). Arrows indicate areas of mucin gene expression. Magnification 200X.

**Figure 2:**
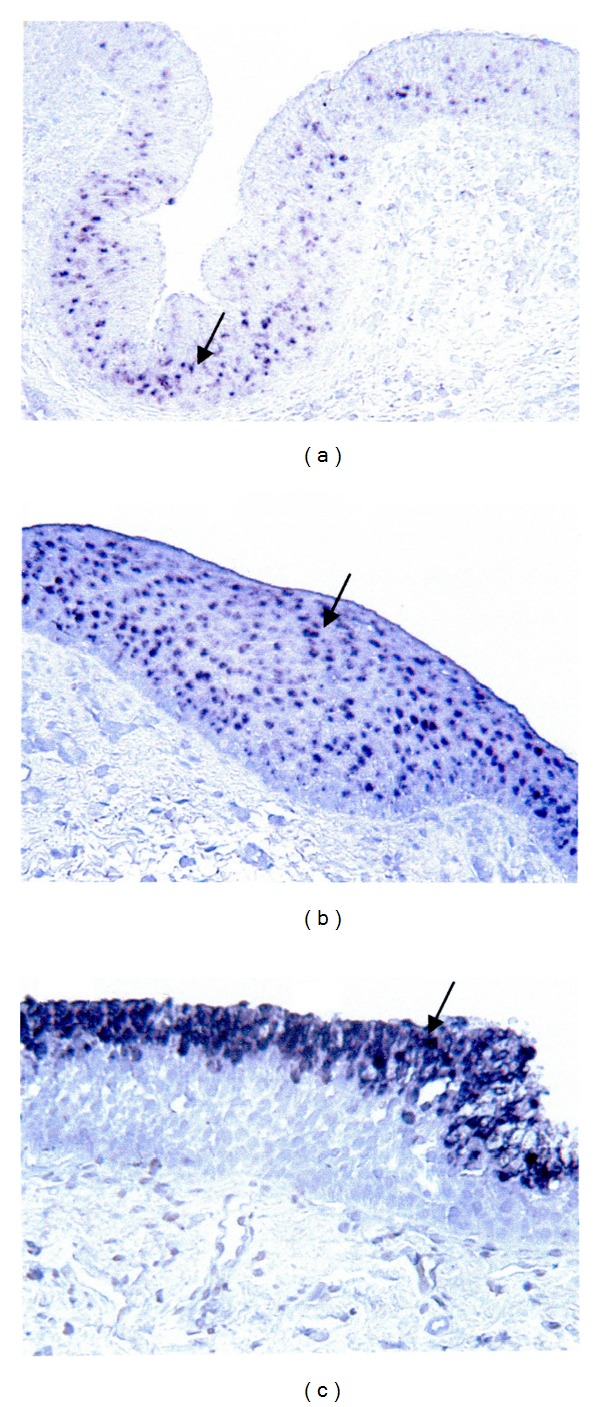
Mucin gene expression in laryngopharyngeal reflux mucosa.* In situ* hybridization photographs of the expression of MUC3 in laryngeal ventricle (a), MUC4 in posterior commissure (b), and MUC5AC in laryngeal ventricle (c). Sections were lightly counterstained in Harris' haematoxylin and then immersed in Scott's modified tap water (bluing reagent). Arrows indicate areas of mucin gene expression. Magnification 200X.

**Figure 3:**
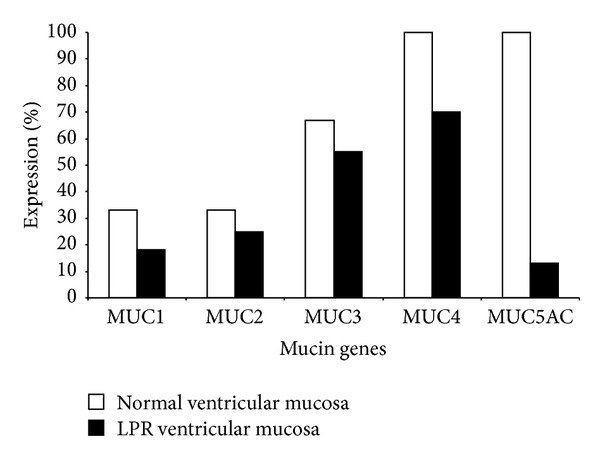
Mucin gene expression in ventricular mucosa of control and laryngopharyngeal reflux mucosa.

**Table 1:** Histological types of reflux mucosa from three laryngeal locations.

Mucosa	Respiratory only		Squamous only		Mixed	
	Control	LPR	Control	LPR	Control	LPR
Posterior commissure	0/3	0/27	1/3	18/27	2/3	9/27
Ventricles	3/3	17/22	0/3	0/3	0/3	5/22
Vocal folds	0/3	00	0/3	14/22	3/3	8/22

Total	3/9 33%	17/71 24%	1/9 11%	40/71 56%	5/9 56%	14/71 20%

The quoted numbers indicate how many patients from whom laryngeal mucosal samples were taken.

**Table 2:** *In situ* hybridisation results of mucin gene expression in the control laryngeal mucosa from 3 control cases.

Mucosa	MUC1	MUC2	MUC3	MUC4	MUC5AC
Posterior commissure	0/3	0/3	2/3	2/3	2/3
Ventricles	1/3	1/3	2/3	3/3	3/3
Vocal folds	0/3	0/3	2/3	2/3	1/3

Total %	1/9 11%	1/9 11%	6/9 67%	7/9 78%	6/9 67%

Samples were taken from 3 laryngeal mucosal samples from each control case and the total was taken for each area for each mucin gene.

**Table 3:** Mucin gene expression in control versus reflux laryngeal mucosa.

Laryngeal mucosa	MUC1		MUC2		MUC3		MUC4		MUC5AC	
	Control	LPR	Control	LPR	Control	LPR	Control	LPR	Control	LPR
Posterior commissure	0/3	4/26	0/3	6/26	2/3	15/26	2/3	17/24	2/3	3/24
Ventricle	1/3	4/22	1/3	5/20	2/3	11/20	3/3	14/20	3/3	3/24
Vocal folds	0/3	7/22	0/3	3/22	2/3	9/21	2/3	14/21	1/3	5/21

Total/average	1/9	15/70	1/9	14/68	6/9	35/67	7/9	45/65	6/9	11/69
	11%	21%	11%	29%	67%	52%	78%	69%	67%	16%

Numbers of mucosal samples for each area of laryngeal mucosa expressing the mucin gene in question are divided by total numbers of samples studied and resultant % calculated. LPR: laryngopharyngeal reflux mucosa.
